# Primary anorectal malignant melanoma treated with neoadjuvant chemoradiotherapy and sphincter-sparing surgery: A case report

**DOI:** 10.3892/ol.2014.1925

**Published:** 2014-02-28

**Authors:** MENG SU, LUCHENG ZHU, WENHUA LUO, HANGPING WEI, CHANGLIN ZOU

**Affiliations:** Department of Radio-Chemotherapy Oncology, The First Affiliated Hospital of Wenzhou Medical University, Wenzhou, Zhejiang 325000, P.R. China

**Keywords:** primary anorectal malignant melanoma, neoadjuvant chemoradiotherapy, sphincter-sparing surgery

## Abstract

Primary anorectal (PA) malignant melanoma (MM) is a rare disease associated with a high mortality rate. The most appropriate treatment strategy for PAMM remains controversial. A 55-year-old female patient, who was misdiagnosed with locally advanced rectal carcinoma, was treated with preoperative radiotherapy and concurrent oral capecitabine. During the therapy, grade 1 leukopenia occurred, however, there was no interruption to treatment. Following chemoradiotherapy, a computer tomography scan identified that the tumor had shrunk significantly and the original enlarged lymph nodes had disappeared. Eight weeks after completion of chemoradiotherapy, sphincter-sparing surgery was performed on the patient and based on the postoperative pathological result, MM was diagnosed. At the time of writing, the patient has survived disease-free for 15 months and at the most recent follow-up examination the Karnofsky Performance Scale score was 100. The therapeutic regimen of neoadjuvant concurrent chemoradiotherapy together with sphincter-sparing surgery is considered to be an optimal choice for patients with PAMM. However, further studies are required to evaluate the efficacy and clinical utility of this therapeutic regimen.

## Introduction

Malignant melanoma (MM) arising from the anorectal region is rare, comprising only 0.4–1.6% of all melanomas and ~1% of all anal canal tumors (1,2). However, the anorectum is the third most common site for the occurrence of primary MM, preceded only by the skin and eyes (3). Occasionally, primary anorectal (PA) MM is misdiagnosed as anorectal cancer, which is attributed to a lack of immunohistochemical findings. As a result, there is no consensus on the most effective therapeutic regimen for PAMM. Preoperative concurrent chemoradiotherapy (CCRT) has been widely used for the treatment of numerous malignant neoplastic diseases, however, not in PAMM. The present case report proposes a novel therapeutic regimen for PAMM by demonstrating a patient who received preoperative CCRT and sphincter-sparing surgery. Patient provided written informed consent.

## Case report

**Figure 1:**
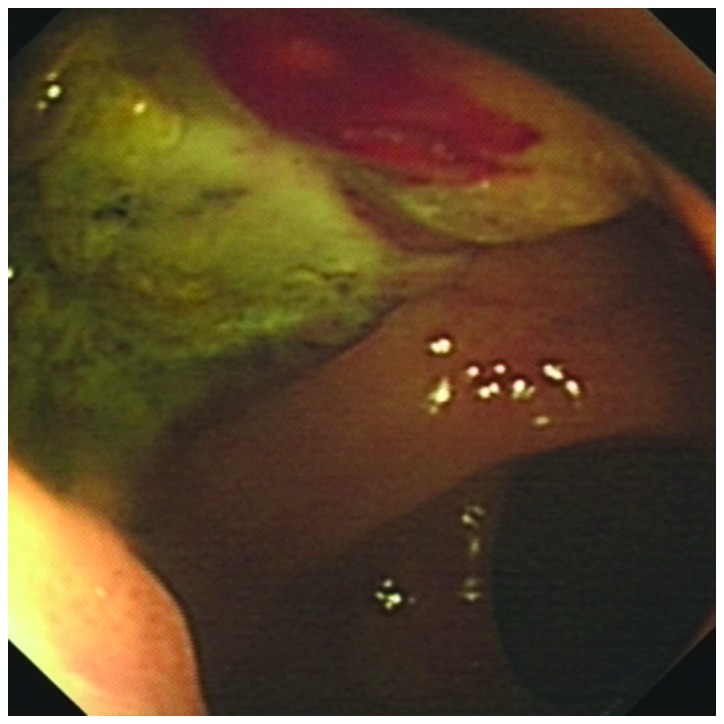
Colonoscopy examination prior to chemoradiotherapy.

A 55-year-old female patient experienced intermittent hematochezia for one year. Digital rectal examination revealed a hard cauliflower-shaped mass (size, 3.0×4.0 cm), located 1 cm from the anal verge, with bloodstains observed on the finger-cot. The serum levels were as follows: Carcinoembryonic antigen, 2.6 μg/l; carbohydrate antigen 19-9, 25.6 U/ml; and lactate dehydrogenase, 180 U/l, which were all considered to be within the normal ranges. Abdominal magnetic resonance imaging (MRI) demonstrated a rectal mass (diameter, 4.2 cm), which had invaded the anus and cervix, in addition, the lymph nodes of the right pelvic cavity and right inguen were enlarged. Through colonoscopy, a pigmented mass with a large ulcer was observed on the anterior wall of the anorectum, ~4.0 cm in size (Fig. 1). The result of colonoscopy pathology indicated low differentiated carcinoma, however, immunohistochemical analysis was not performed. According to the abovementioned findings, the preoperative diagnosis was a locally advanced rectal carcinoma. In order to preserve sphincter function, obtain pathological downstaging and reduce the rate of recurrence, preoperative CCRT was adopted.

A total irradiation dose of 45.0 Gy was delivered in daily fractions of 1.8 Gy, five times per week, through a pair of opposed anterior-posterior fields using a 6-MV linear accelerator. The treatment fields were set as follows; the superior border was placed at S1, the inferior border was placed below the anus (~0.5 cm) and the lateral borders of the planning target volume were 1.5-cm lateral to the widest bony margin of the true pelvic wall. Throughout the radiation period, capecitabine was administered every 12 h, twice a day following a meal, at a dose of 1,000 mg for two weeks. Administration of the medication subsequently ceased for one week. Following this, the medication regimen was continued for another two weeks. During the therapy, only grade 1 leukopenia occurred and the treatment was well-tolerated by the patient, with no regimen interruption.

A digital rectal examination, following CCRT, revealed a hard cauliflower-shaped mass (size, 2.0×2.0 cm) without any bloodstaining observed on the finger-cot. The computer tomography scan demonstrated that the tumor had shrunk sharply (diameter, 2.0 cm) and there were no enlarged lymph nodes.

**Figure 2:**
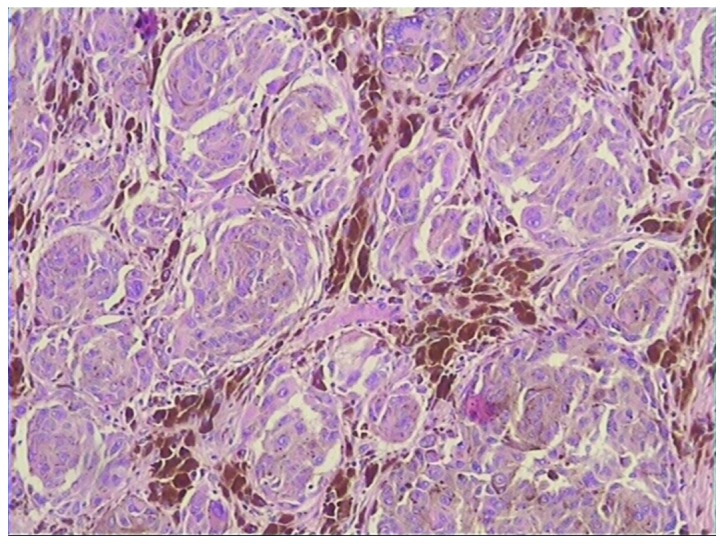
Histopathological examination demonstrated numerous melanin granules between the tumor cells (hematoxylin and eosin staining; magnification, ×400).

The surgery was performed eight weeks following the completion of CCRT. A cauliflower-shaped tumor (size, 2.0×2.0 cm), which invaded the posterior wall of the vagina, was observed on the antetheca of the anal region. However, the enlarged lymph nodes of the right pelvic cavity and right inguen, which were observed in the prior MRI scan, were no longer detected. The rectum, uterus, bilateral accessory and posterior wall of the vagina were excised during surgery. Finally, coloanal anastomosis and reconstruction of the vagina were performed. A histopathological examination demonstrated cells, which exhibited marked cytological atypia, pleomorphism and increased mitotic activity. Numerous melanin granules were apparent between the tumor cells (Fig. 2). Immunohistochemical staining for HMB-45 (weakly positive), Melan-A (positive), S-100 (weakly positive) and VIM (weakly positive) confirmed the diagnosis of PAMM. According to the American Joint Committee on Cancer classification for melanoma (7th edition) (4), the postoperative stage was identified as ypT2aN0M0, stage IB, while the initial stage was cT4bN2M0, stage III. In addition, the resection margins were negative. There was no complication following the surgery and the postoperative recovery was good. The patient did not receive any subsequent adjuvant therapy.

The patient underwent a close postoperative follow-up for 15 months, without any evidence of local relapse, lymphatic metastasis or distant metastasis. As a result of this treatment strategy, the patient obtained complete remission with a high quality of life. The only symptoms experienced by the patient were pain from the incision for one month and occasional instances of constipation in the six months following surgery. Recently, the patient has not complained of any symptoms or signs. In the postoperative follow-up period, the mean Karnofsky Performance Scale (KPS) score was 90 and the most recent score was 100.

## Discussion

MM is a neoplasm of neuroectodermal origin arising from melanocytes. PAMM most commonly originates near the anorectal junction, where melanocytes normally occur. Goldman *et al* (5) reported 49 cases of PAMM, and 45 of those were located at, or near, the anorectal junction. As MM arising from the anorectum was initially described by Moore in 1857 (6), a series of articles describing PAMM have been published. The incidence remains extremely low, ranging between <1 and 3% (7). In addition, PAMM is a malignant disease with an exceptionally poor prognosis. The five-year survival rate is only 9–16% (8,9), which may be explained by the aggressive nature of the disease. A previous study identified that 26% of patients had exhibited distant metastasis at the time of diagnosis (10).

PAMM can easily be mistaken for certain other lesions, including hemorrhoids, polyp and adenocarcinoma, and misdiagnosis rates have been recorded as 58.2–86.4% (9,11,12). There are several common causes for misdiagnosis in PAMM, including i) the symptoms and signs of PAMM are not specific; ii) non-pigmented lesions appear on endoscopic examination; iii) immunohistochemical examination as a definite diagnosis approach is not performed in every colonoscopy pathology; and iv) MM located on the anorectum is rare, and inexperienced physicians lack the vigilance and awareness of this disease.

In the present case, a pigmented mass was revealed on the colonoscopy, however, the main cause of misdiagnosis was that an immunohistochemical examination was not performed. Misdiagnosis of PAMM may lead to a delay in treatment and distant metastasis. In one study, the five-year survival rate of misdiagnosed patients was 11%, compared with 25% that had been diagnosed correctly (9).

As there is a low incidence of, and inexperience with treating PAMM, the most appropriate treatment strategy remains controversial. Surgery as a primary treatment strategy for PAMM, ranges from abdominoperineal resection (APR) to wide local excision (WLE). However, previous studies comparing the difference in survival time of patients treated by APR or WLE identified no statistical difference (10,13). In a number of studies, adjuvant chemotherapy (14), adjuvant radiation (15), neoadjuvant radiation (16) and immunotherapies (17) have also been used in PAMM, and certain curative effects were obtained. Preoperative CCRT has a proven role in the treatment of various malignant diseases, particularly in locally advanced rectal cancer (18). The predominant role of preoperative CCRT is to reduce locoregional and pelvic cavity recurrence, and to obtain a higher rate of sphincter preservation via tumor shrinkage. In addition, it facilitates the removal of potential micrometastasis and lessens distant metastases. In the present case, preoperative CCRT was performed in PAMM and the patient achieved a pathological partial response. Uner *et al* (16) reported a case of PAMM; the patient received radiation as the primary treatment rather than surgery and exhibited a similar outcome, indicating that neoadjuvant therapy may be beneficial to PAMM. The goal of therapy for PAMM should be to maximize the survival time as well as to improve quality of life. Preservation of sphincter function is, therefore, emphasized in surgery. Following CCRT, a decrease in tumor volume renders sphincter preservation feasible with negative margins. The timing of surgery following CCRT should also be considered. In the period following radiation therapy, tumor cells may be eradicated continually, and the adequate time between CCRT and surgery is 4–8 weeks (17).

KPS was a tool, developed as a subjective means of evaluating a patient’s ability to perform ordinary tasks. It is a clinical prognosis index of the cancer patient population. In the current case, the patient exhibited a high KPS score following surgery, which indicates a good prognosis.

In conclusion, PAMM remains a rare disease that is associated with a poor prognosis. In order to improve the accuracy of diagnosis, physicians must be vigilant to the occurrence of PAMM, and immunohistochemistry should be a routine examination during colonoscopy pathology. The most effective treatment strategies for PAMM remain the focus of investigation. The therapeutic regimen of neoadjuvant CCRT together with sphincter-sparing surgery may guarantee a high quality of life and provide high efficacy, indicating that it may be an optimal strategy for patients with PAMM. Further studies are required to further evaluate the efficacy of this therapeutic regimen.
